# Naturalization and invasion potential of *Sesuvium portulacastrum* L. recorded as alien species in Egypt

**DOI:** 10.1038/s41598-024-53627-7

**Published:** 2024-02-07

**Authors:** Selim Z. Heneidy, Laila M. Bidak, Marwa Waseem A. Halmy, Amal M. Fakhry, Soliman M. Toto, Eman T. El Kenany

**Affiliations:** 1 Department of Botany and Microbiology, Faculty of Science, Alexandria University, Alexandria, 21511 Egypt; 2 Department of Environmental Sciences, Faculty of Science, Alexandria University, Alexandria, 21511 Egypt; 3 Department of Oral Biology, Faculty of Dentistry, Pharos University in Alexandria, Alexandria, Egypt

**Keywords:** Ecology, Environmental sciences

## Abstract

*Sesuvium portulacastrum* is a perennial halophyte of family Aizoaceae, non-native to Egypt, which was introduced from France ten years ago as an ornamental species. This study reports the detection of *S. portulacastrum* in the wild in Egypt. Voucher specimens were deposited in the Herbarium of Alexandria University (ALEX). A population of the species was recorded in the wild near Maruit Lake in the north-western coast of Egypt in 2018 during plant resources surveys of the region. The study aimed to assess the potential for *S. portulacastrum* to spread as an alien species through field observations and geospatial measurements under current conditions in its new habitat. The measured morphological parameters were higher than those recorded in its native habitats. The field observation for three years revealed that the species is proliferating and expanding in the investigated site forming large mats of mean size of up to 9 m^2^. The spatial extent of *S. portulacastrum* based on the EOO and AOO was quantified, and the expansion rate was estimated at 0.16 ha/year in the investigated site. The geospatial parameter used in the study will not only help in determining the spread rate of the alien species spatially and temporally, but also in its effective management through guiding managers in developing monitoring plans for the species under the changing climate uncertainty. Continuous monitoring and early detection of any potential threats of the introduced species are highly recommended, to avert any potential adverse impacts on native biodiversity and assess its behaviour in the wild habitat.

## Introduction

Invasive species are deemed as one of main threats to biodiversity globally^1^. Accidental introductions are the key cause of invasive plants worldwide. The number of introduced species, particularly plant species is projected to increases with the growth in global trade, travel, ease of communication, and advancement in transportation. A considerable proportion of the introduced species may turn invasive in the new habitats causing serious socioeconomic and ecological impacts^2^. Thus, understanding the mechanisms through which introduced species turn into invasive that may seriously influence native biodiversity is a priority. Monitoring of introduced species is critical for understanding their dynamics in new habitats, and to help guide management activities directed towards controlling their expansion^3^. The authors came across *Sesuvium portulacastrum* L. as an unexpected record during field surveys of the flora of the northwestern coastal region of Egypt in 2018. The species is not native to Egypt and was recorded for the first time in the Wild. The plant has not been recorded as part of the floral composition of the north-western coastal region at any previous study (e.g.,^4–10^), including all the study projects that conducted long term monitoring of the flora of the region (e.g.^11,12^), and sustainable use of wild medicinal plants,^13,14^. It is established that if a species has not been recorded in the flora of an area for over 50 years, and later detected then it will be considered as a new record species (personal communication Adel Al- Gazzar). According to this concept *Sesuvium portulacastrum* is considered as a new record species in Egypt generally and in the northwestern coast specifically. No records available for the species in the flora literature, and it has not been recorded in any of flora records of Egypt for more than 50 years. The authors did not recognize any records and/or observations for the *S. portulacastrum* in the previous studies on the Egyptian flora or vegetation composition. New records of the species in the central and southern regions of neighboring Saudi Arabia have been detected by^15^.

The field survey conducted in the region has led to the detection of the species unknown to the Flora of Egypt in the calcareous sandy plains of the region. Therefore, the current study aims at (1) reporting the occurrence of *S. portulacastrum* as an alien species to the flora of Egypt. The detailed morphological description, distribution and habitat of the species are provided supported by photographs taken in the field. (2) The study aims also at assessing the status of the species in the new habitat in the north-western coastal region of Egypt; and (3) evaluating the potential economic and ecological value of the species in the new habitat.

### The study species

*Sesuvium portulacastrum* L. is a perennial creeping herb belongs to family Aizoaceae^16^. The dicotyledonous plant is commonly called sea purslane or shoreline purslane^17,18^). The species was first named by Linnaeus as *Portulaca portulacastrum*, he later moved it to *Sesuvium*^19^. The species is not known to occur in Egypt, however^4^ reported the annual *Trianthema portulacastrum* L. (syn. *Sesuvium portulacastrum* L.) as very rare wild species restricted in its distribution to Gebel Elba. The species is an important psammophytic facultative halophyte. It has a distinct physiological and molecular elasticity that enables it to adapt and survive under various stress conditions^20–23^.

### Distribution and habitat

*Sesuvium portulacastrum* is one of the most commonly widespread coastal species of Sesuvioideae; the smallest subfamily of Aizoaceae. Members of the subfamily exist mostly in the subtropical regions of Australia and Africa with some species distributed in Asia and the Americas^24^. Generally, members of the subfamily inhabit saline soils or disturbed semi-arid habitats in warm climates^25–27^). *S. portulacastrum* has been recorded on the shorelines of five continents^28,29^. It usually inhabits warm coastal ranges from Mediterranean to sub-tropical regions extending from the equator to nearly 34° north and 42° south^30–33^). The species exists on many tropical islands and commonly as a pioneer on coastal dunes and coastlines in tropical and sub-tropical regions^17,24,34,35^.

The plant is common on a variety of substrates including limestone-shell, coral, pyroclastic sand shingle, and unconsolidated beaches;^27,30,36^. It prefers growing in wet sandy areas including beaches, coastal dunes, mangroves, salt flats and marshes. However, the species can withstand drought and salinity, and can inhabit low annual rainfall and long dry seasons areas, in addition to areas affected by salt spray deposition^37^. It grows widely on the edges of the hurricane wash over channels, tidal flats, and disturbed roadsides^36,38,39^.

The species can withstand the continuous sand movements triggered by prevailing winds on coastlines. It traps sand and tolerates burial by accreting sand. The shoots, remain fleshy after burial^17^. *S. portulacastrum* is also reported as an important sand binding species on heavy siliceous sand, including heavy sands derived from volcanic rocks, limestone coastal dunes and mangrove habitats^41^. The species exists in the Great Barrier Reef, Australia, on calcareous coral, coral sand and on mixture of coral fragments and coral sand^42^. However, it was reported that the species does not occur on pure coral sand^43^.

### Economic and ecological importance

The plant is salt tolerant, drought resistant that can be used for bio-reclamation of saline soil in the arid and semiarid regions through the phyto-desalinzation. It can also be used in rehabilitation projects, as an alternative culture to problematic soils, and in land reclamation of saline soil. The species has been reported to be useful as ground cover plant to avoid erosion in dunes. *S. portulacastrum* has been also reported to be commonly used in environmental protection particularly for sand dune fixation, saline soil stabilization and desalination, as well as in landscaping as ornamental plant^37,44^. The use of the species as an ornamental plant is recommended as a ground cover plant in a sandy, well-drained soil in the full sun conditions. Particularly, as the plant requires minimal irrigation and fertilizer inputs once it is established in the landscape. In other words, it can be defined as a low maintenance ornamental plant. However, it should be noted that the plant is not suited for growing in shady areas^45^. The species has extraordinary capability to endure various stress conditions^33^, as it can tolerate windy and salty conditions common in coastal regions and can help build dunes by catching sand in between stems and leaves^17^.

Moreover, *S. portulacastrum* has been reported as an edible plant that is consumed occasionally in many countries as a vegetable by the local people^17,37^. The plant is grown as a vegetable and used for cooking purposes in Asia, particularly in India and Southeast Asia^46^. It has a great potential nutritive value and utilized as a wild vegetable crop in the southern India because of its salty taste and fleshy nature^32,33^). The species has also a grazing value as it has been reported as one of the species used as feed for domesticated animals in its native range. Several animals reported to graze on it, for example, jack rabbits, sheep, goats, camels, and has been reported as a favourite food of crabs^17,37^. When compared to common fodder, it appears that the plant has high nutritional composition (Table 1). This reveals the potential for using the plant as a fodder species. Growing the plant in the arid and semiarid regions can provide a potential alternative source of fodder to domesticated animals^47^. The species has been reported as an important medicinal plant used in folk medicine in its native range, especially the leaves which has been used as antiscorbutic^48^. The species is used as a haemostatic and the decoction is considered the best antidote for stings of venomous fish^33^).

**Table 1:** Mean percentage of total digestible nutrients (TDN) and digestive crude protein (DCP) in supplementary feed and the study species (source:^37,49,50^).

Species	TDN	DCP
*Triticum vulgare* Vill. (Wheat)	62.23	5.32
*Hordeum vulgare* L. (barely)	64.00	5.98
*Zea mays* L. (corn)	68.00	3.98
*Trifolium alexandrinum* L. (clover)	56.00	11.8
*Sesuvium portulacastrum L.*	55.14	5.97

## Materials and Methods

### Study area

**Figure 1:**
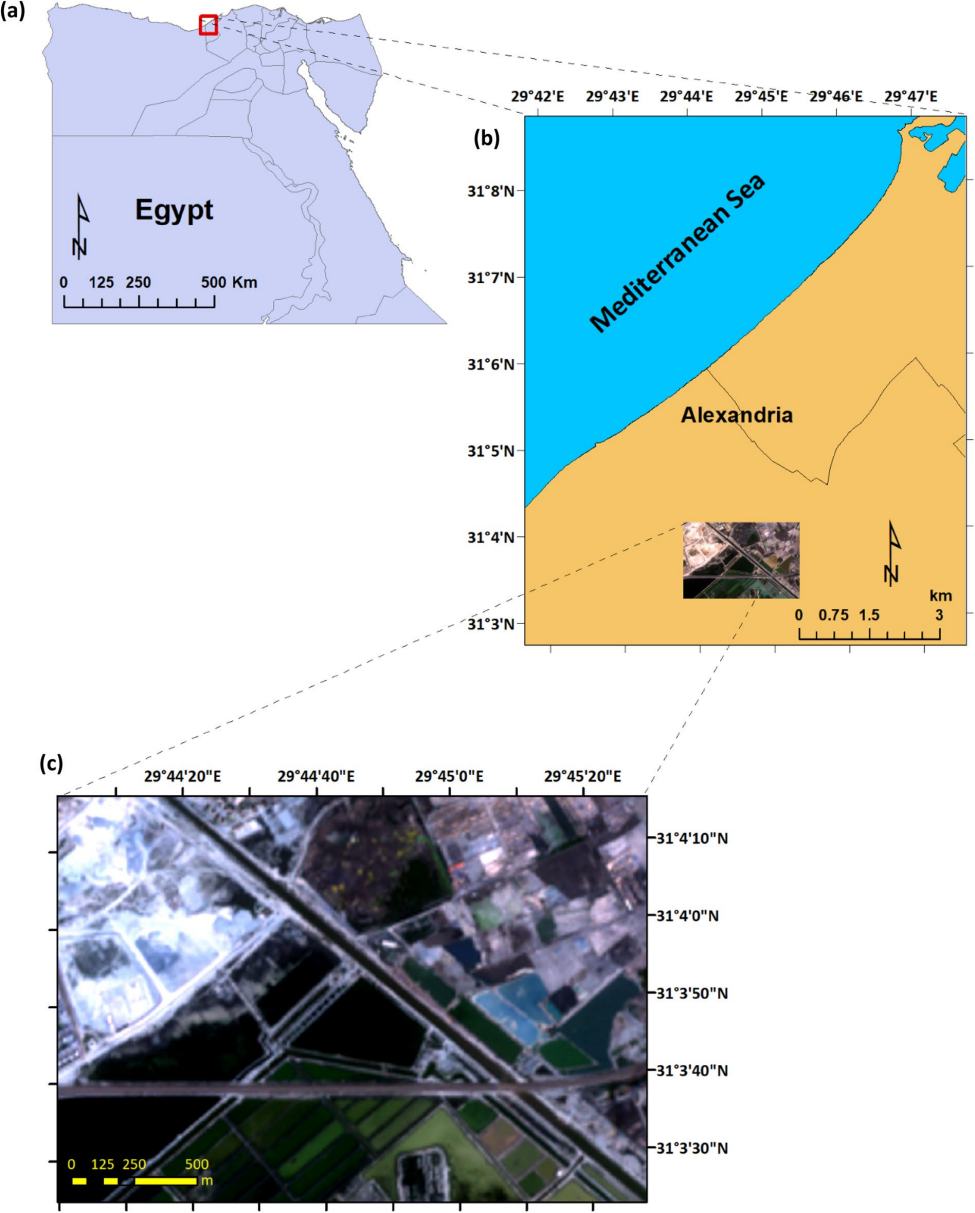
Location of the study area in Egypt (**a**). Part of the northwestern coast of Egypt (**b**), containing an inset subset of a Sentinel 2 satellite image that illustrates the region where *S. portulacastrum* was recorded; (**c**) the enlarged view of the subset of the satellite image acquired on 8/3/2018 for the study area.

The study area is part of Maruit region located at the north-western Mediterranean coastal strip of Egypt (Fig. 1). The area represents the sandy saline habitats bordering Lake Mariut, which is mainly dominated by halophytic plants.

**Figure 2:**
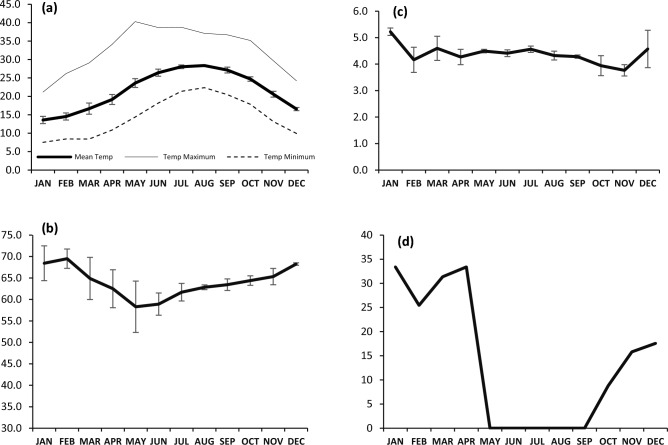
The monthly mean of the climatic parameters (**a**) temperature (°C); (**b**) relative humidity (%); (**c**) wind speed (m/s); and (**d**) precipitation (mm) for the study area averaged over the period from 2018 to 2020.

 Climatically the region lies in Meigs's area of ‘warm coastal deserts’^51^ characterized by warm summer with a mean temperature less than 30 °C (Fig. 2). Rainfall in the region area is scanty, irregular, variable and restricted to few months from late autumn to early spring. Not only the annual rainfall shows variations, but also the amount of rain in the corresponding months varies in the successive years. Though occasional short rainstorms can occur in winter, most of the days are sunny and mild. The relative humidity is high can reach 65% or more in summer, but much lower in winter and decreases with distance landward. The months with low humidity are those during the blowing of the hot Khamasin winds mainly in April and May. The main habitat types in the region include calcareous sand dunes of saline soils and saline depressions^52^.

### Field survey and data collection

The species was first recorded on the banks of an irrigation canal near Maruit Lake at the north-western coast of Egypt in 2018. Successive field surveys for monitoring of the species and collections of plant material were conducted at different time periods between 2018 and 2020. Field trips were organized at intervals to ensure collection of plant material at different phenological stages especially at flowering and fruiting stages. Plant material was collected in accordance with the applicable national and international guidelines of the IUCN^53^ and the Convention on the Trade in Endangered Species of Wild Fauna and Flora. The permission for the collection of the plant species for scientific purposes was obtained from The Department of Botany and Microbiology at the Faculty of Science -Alexandria University. Plant specimens collected were processed to make herbarium specimens that were then identified and deposited as voucher specimens in the Herbarium of Alexandria University (ALEX) at the Faculty of Science, and at the herbarium collection of Alexandria University Botanic Garden (Heneidy et al. collection, serial no. 5501-5503). ALEX is a public herbarium providing access to the deposited material.

The identification of plant specimens was conducted by Professor Dr. Adel El-Gazzar according to the available sources, and floras including^4,5^, in addition to consulting other sources (e.g.,^54,55^). Several visits were made to Egyptian herbaria to check for the existence of *S. portulacastrum* (e.g., Assiut University (ASTU), Ain Shams University (ASUE), South Valley University (ASW), Cairo University (CAI), the Desert Research Center, Mataria (CAIH), the Agricultural Research Center (CAIM), the National Research Centre (CAIRC), Mazhar Botanical Garden (MAZHAR), Suez Canal University (SCUI), and Tanta University (TANE). No preserved herbarium specimens of *S. portulacastrum* was found.

Collected field observations have been recorded to study floristic composition and habitat features including main habitat types, major edaphic features, species habit, colour of flowers, dominant species and all other associated species (Figs. S1–S3, Supplementary Material). Provisional identification was made by the help of^4,9^, in addition to consulting other monographs and revisions.

Stands with 20 m × 20 m size, were selected to study *S. portulacastrum* in the investigated region. Stands were selected to represent the prevailing environmental variations associated with the distribution of the study species as much as possible. Cover percentage was calculated for the plant in the selected stands using line- intercept method^56^.

Five patches of 20 seeds each were weighed to determine the mean seed mass in µg of the *S. portulacastrum*. From the field observations the population size of the species was estimated, and the individuals were classified according to the mat size into large, medium and small.

### Soil analysis

Soil samples were taken from the surface to the depth of 20 cm close to the growing roots of the plants. Soil samples were brought to the laboratory, air dried over sheets of paper, passed through a sieve with a 2-mm mesh size to remove gravels and debris, and then kept in paper bags ready for subsequent analysis. The moisture content was calculated and expressed as moisture percent. Soil water content (SWC) was determined according to the following equation after^57^:

$${\text{SWC }}\left( \% \right) \, = \, \left( {{\text{FW}} - {\text{DW}}} \right)/{\text{DW}} \times { 1}00$$

where FW was the fresh and DW was the dry weight of the soil. Soil was oven-dried at 105°C to constant weight. The soil extract (1:5) was prepared for each soil sample and used for assessing the EC, pH and other parameters. The measurement of the electrical conductivity (EC) was carried out by means of a direct indicating conductivity bridge (ds\m). Soil pH was determined using glass electrode pH meter^58^. The estimation of chlorides was carried out by titration method using silver nitrate according to^59^. Sulphate content was estimated gravimetrically using barium chloride solution^60^. Concentration of sodium was assessed by flame photometer as described by^61^. The soil texture was determined through the Bouyoucos hydrometer following the method described by^62^ and consequently, the soil type was identified using the soil triangular. Determination of CaCO_3_ was carried out using Collin’s calcimeter^63^. The total organic matter was estimated by loss on ignition method at 550 °C according to^64^.

### Morphological and micro-morphological studies

For the purpose of the micro-morphological examination of *S. portulacastrum* in the investigated site and comparing that to the published characteristic in its native range; 8–10 segments of the main stems, each measuring 3–11 mm in diameter were collected from the species under study. The stem segments of ten plants collected from branches adjacent to the ground level were immediately fixed in formalin–acetic acid–alcohol (FAA) according to^65^. Following appropriate trimming process, the processed segments were subjected to dehydration in tertiary butyl alcohol (TBA) series, and then treated using the routine method of paraffin embedding. Transverse sections of 12- to 15-mm thick were obtained from the processed segments, and then microtomed using either a Leica rotary microtome and stained with safranin fast green combination according to^66^; or a tannic acid–ferric chloride–lacmoid mixture following^67^. In addition, the thick stem segments were also processed and sectioned on a sliding microtome.

### Vegetative propagation and seed germination

For testing the seed viability and germination potential of the species, seeds were collected from underneath the plant mats in the investigated site during the successive field visits. The seeds were kept in the seed bank at the Department of Botany and Microbiology-Faculty of Science-Alexandria University. Three replicates of 10 seeds were placed in three petri-dishes, and the seed germination was observed for a week.

Germination was also tested in the soil, where 5 seeds were placed in three pots each 20 cm in diameter filled with clay loam soils. The experiment lasted for two weeks.

The vegetative propagation potential of species was tested by collecting five rooted segments of the main stem each measuring 3–9 mm in diameter and 40–60 mm in length, which were used for vegetative propagation experiment at the Botanic Garden of the Faculty of Science –Alexandria University (Fig. S4, Supplementary Material). The plant segments were planted in well-drained sandy soil and were spaced 75–150 cm apart and the growth of the segments was observed.

### Current distribution and rate of expansion

The locations of *S. portulacastrum* individuals were identified using a global positioning system (Garmin GPSMAP 64sx GPS). The coordinates of these points were included into the geographic information system (GIS) domain and used for assessing the measure of the spatial expansion. Expansion speed of *S. portulacastrum* was assessed here according to the approach discussed in^68,69^, as the increase in area of occurrence (AOO) in the study area over the investigated period, regardless of the processes or ways involved. Natural dispersal, through the active and passive mechanisms, was assumed as the acting process in the assessment area. The use of change in AOO as a generic measure of the spread of alien species is recommended when few years of data exist. The expansion speed of an alien species is assessed based on the measures of the area of occupancy (AOO, the cumulative area of all occupied grid cells) and the extent of occurrence (EOO, the smallest convex polygon encompassing all occurrences)^70^. The assessment of the change in the AOO can be utilized as a measure for the evaluation of the potential invasiveness of introduced alien species (e.g.,^69,71–73^). For the purpose of the current study a grid with 10 m cell size was used for the assessment of AOO at the scale of study area, where a grid with cells each with 10 m × 10 m was placed over study area and the cells where the records of *S. portulacastrum* intersect were determined within the defined area of study. For the assessment of AOO at the national scale a grid with cells each with 2 km × 2 km was placed over the layer of Egypt and the cells where the records of *S. portulacastrum* overlap were defined.

The approach followed requires few years of data for assessment of the expansion speed (*ES*) of alien species as follows:

$$ES = \frac{\sqrt{{AOO}_{t}}- \sqrt{{AOO}_{t-\tau }}}{\sqrt[\tau ]{\pi }}$$

where AOO_t_ is defined as the AOO in year _t_; and τ is the number of years between the start earliest date and latest date for the estimation of AOO.

The expansion speed was calculated at two scales; the study area local scale and the national scale. For the assessment of the change in the EOO at the study area and national scale over the investigation period, the convex hull bounding the observation records in the successive years was estimated by the minimum bounding geometry tool in ArGIS 10.1^74^. The global occurrence record data of the species were obtained from GBIF in 2018, 2019, and 2022^75,76^, and were used to create global distribution map of the species occurrence and track the change of the records of *S. portulacastrum*. All the maps were produced within the framework of the GIS software package ArcMap 10.1^74^.

## Results

### Ecological monitoring of the species

The plant was found on saline soils that ranged in texture from sandy to sandy-loam (Table 2) on the banks of an irrigation canal, which has been drilled about ten years ago, near Maruit Lake in the northwestern coastal desert of Egypt (Fig. 1). It is growing as a runner succulent, forming large mats. The mean mat size of individuals ranged from 6.75 ± 1.00 m^2^ in 2018 to 8.07 ± 1.21 m^2^ (Table 3). The average mat dimension ranged from 362.4 ± 89.65 cm × 280.6 ± 33.41 cm in 2018 to 418.03 ± 94.80 cm × 290.55 ± 38.39 cm in 2019. In general, the averaged mat dimension of young individuals was 185.05 ± 43.55 × 129.25 ± 28.35 cm.

**Table 2:** The physical and chemical characteristics of soil samples obtained from the designated sites where the *S. portulacastrum* populations were recorded include soil texture (percentage of sand, silt, and clay), organic matter and calcium carbonate (CaCO_3_) percentage, electrical conductivity (EC in dS/m), and the concentrations of sodium (Na), chloride (Cl), and sulfate (SO_4_^2–^) measured in Meq/l.

Site	Soil texture			Soil Type	OM%	pH	EC (dS/m)	Na^+^ (Meq/l)	Cl^−^ (Meq/l)	SO_4_^2–^ (Meq/l)	CaCO_3_%
	Sand%	Silt%	Clay%								
1	73.52	14	12.48	Sandy loam	0.14	8.6	122.4	1652	299	272.9	15
2	81.52	10	8.48	Loamy sand	0.72	8.3	122	1565	299	367.5	5.2
3	83.52	6	10.48	Loamy sand	0.21	8	30	260.87	257	46	10.5
4	92.32	−	7.68	Sandy	0.14	7.2	1.14	2.39	3.22	6.69	15.9

**Table 3:** Parameters measured for the *S. portulacastrum* population recorded in the north-western coastal desert of Egypt in 2018 and 2019.

Measured parameters	Mean ± SD	t-statistic	p-value
Mat dimensions (cm)			
2018	362.4 ± 89.65 × 280.6 ± 33.41	1.0229	0.3237
2019	418.03 ± 94.80 × 290.55 ± 38.39		
Mat area (m^2^)			
2018	6.75 ± 1.00	1.6818	0.1436
2019	8.07 ± 1.21		
Cover (%)	3.87		
Stem diameter (cm)	6.7 ± 4.00		
Mean branch length (cm)	121.5 ± 35.56		
Mean number of flowers per branch	43 ± 6.84		
Seed mass (µg/20 seeds)	10.58 ± 0.72		

The runner stolon are terete glabrous succulent and greenish pink or red in colour, it can branch to 2.0 m, but usually shorter with a mean length of 121.5 ± 35.56 cm. They are diffusely branched with oppositely arranged simple, petiolate succulent leaves up to 3 cm in length and the petiole up to 2 mm. Leaves are characterized by reddish green colour with entire margin and acuminate apex. The stems are photosynthetic becoming reddish with age. The plant holds a tap root system; however, due to its creeping nature, it irregularly produces adventitious roots from the nodal region. The plant produces commonly pink to purple solitary flowers and blooms throughout the year. Flowers are incomplete, actinomorphic with mean length of 1.5 cm. The calyx has five pink sepals. There is no corolla. Stamens are numerous. Ovary is superior, 3–5 locules, ovules numerous, styles 3–5 each up to 3 cm in length. Capsule ellipsoid/ovoid, membranous, dehiscent enclosing small smooth black seeds that range from 1.2 to 1.5 mm in length.

The mean cover percentage of *S. portulacastrum* in the study area was estimated as 3.87%. The mean recorded population size of *S. portulacastrum* was 84 individuals. A total of 30 plant species were recorded as associated with *S. portulacastrum* population in the study site (Table 4), of which 14 were perennials and 16 were annuals. Those species are belonging to eleven families. About 70.0% of them are belonging to three families Poaceae, Chenopodiaceae and Asteraceae (30.0, 23.3, and 16.7% respectively). The most commonly associated species with *S. portulacastrum* were *Arthrocnemum glaucum* (a halophytic perennial grows in salt places)*, Phragmites australis* (a perennial grass grows in wetland*s*)*, Tamarix nilotica* (woody perennial grows especially in salty places) and *Mesembryanthemum forsskali* (succulent annual herb).

**Table 4:** List of species found associated with *S. portulacastrum* population recorded in the studied site.

Species	Family
Perennials	
*Arthrocnemum glaucum* (Del.) Ung.Sternb*	Chenopodiaceae
*Atriplex halimus* L.	Chenopodiaceae
*Convolvulus arvensis* L.	Convolvulaceae
*Cynodon dactylon* (L.) Pers	Poaceae
*Enarthrocarpus lyratus* (Forssk.) DC	Brassicaceae
*Halocnemum strobilaceum* (Pall.) M. Bieb	Chenopodiaceae
*Juncus rigidus* Desf	Juncaceae
*Launaea nudicaulis* (L.) Hook. f	Asteraceae
*Limoniastrum monopetalum* (L.) Boiss	Plumbaginaceae
*Phragmites australis* (Cav.) Trin.ex Steud*	Poaceae
*Salsola longifolia* Forssk	Chenopodiaceae
*Suaeda pruinosa* Lange	Chenopodiaceae
*Tamarix nilotica* (Ehrenb) Bge*	Tamaricaceae
*Zygophyllum album* L.	Zygophyllaceae
Annuals	
*Avena fatua* L.	Poaceae
*Bassia muricata* (L.) Murr	Chenopodiaceae
*Bromus rubens* L.	Poaceae
*Cakile maritime* Scop	Brassicaceae
*Chenopodium murale* L.	Chenopodiaceae
*Cutandia dichotoma* (Forssk)Trabut	Poaceae
*Lolium perenne* L.	Poaceae
*Malva parviflora* L.	Malvaceae
*Mesembryanthemum* forsskali Hochst*	Aizoaceae
*Parapholis marginata* Runemark	Poaceae
*Phalaris minor* Retz	Poaceae
*Reichardia tingitana* (L.) Roth	Asteraceae
*Schismus barbatus* (L.) Thell	Poaceae
*Senecio desfontainei* Druce	Asteraceae
*Sonchus oleraceous* L.	Asteraceae
*Urospermum picroides*(L.) F.W.Schmidt	Asteraceae

*Most common associated species.

### Current distribution and rate of expansion

**Figure 3:**
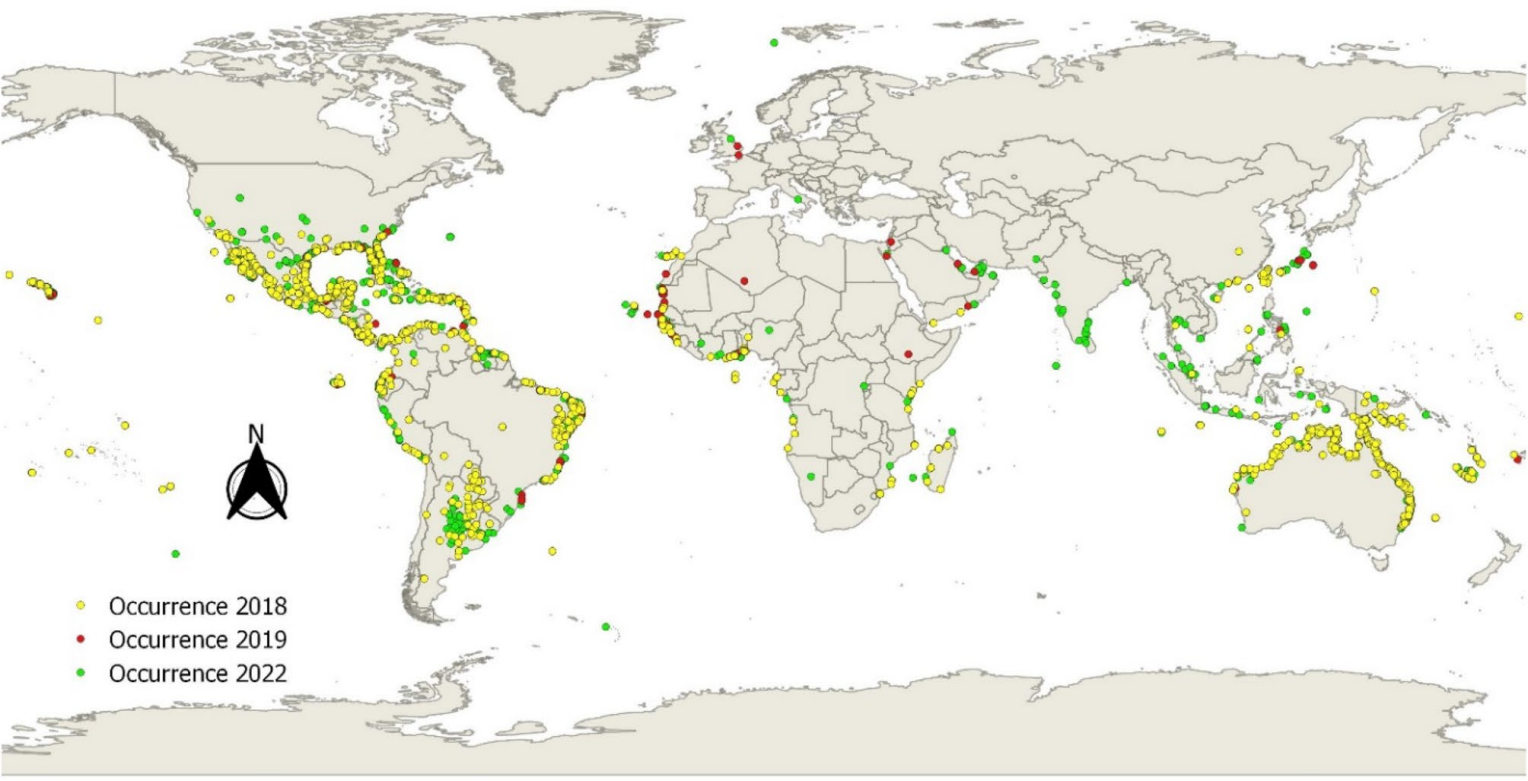
Global distribution of occurrence records of *S. portulacastrum* based on data obtained from GBIF in 2018, 2019, and 2022^75,76^. The map was produced within the framework of ArcMap 10.1 GIS software package^74^.

The global distribution of the occurrence records of *S. portulacastrum* obtained from GBIF (Fig. 3) revealed a trend towards expansion in the species distribution in areas outside the native range of the species between 2018, and 2022. More records were detected in the recent years in areas in which the species is not native, particularly in North Africa and West Asia.

**Figure 4:**
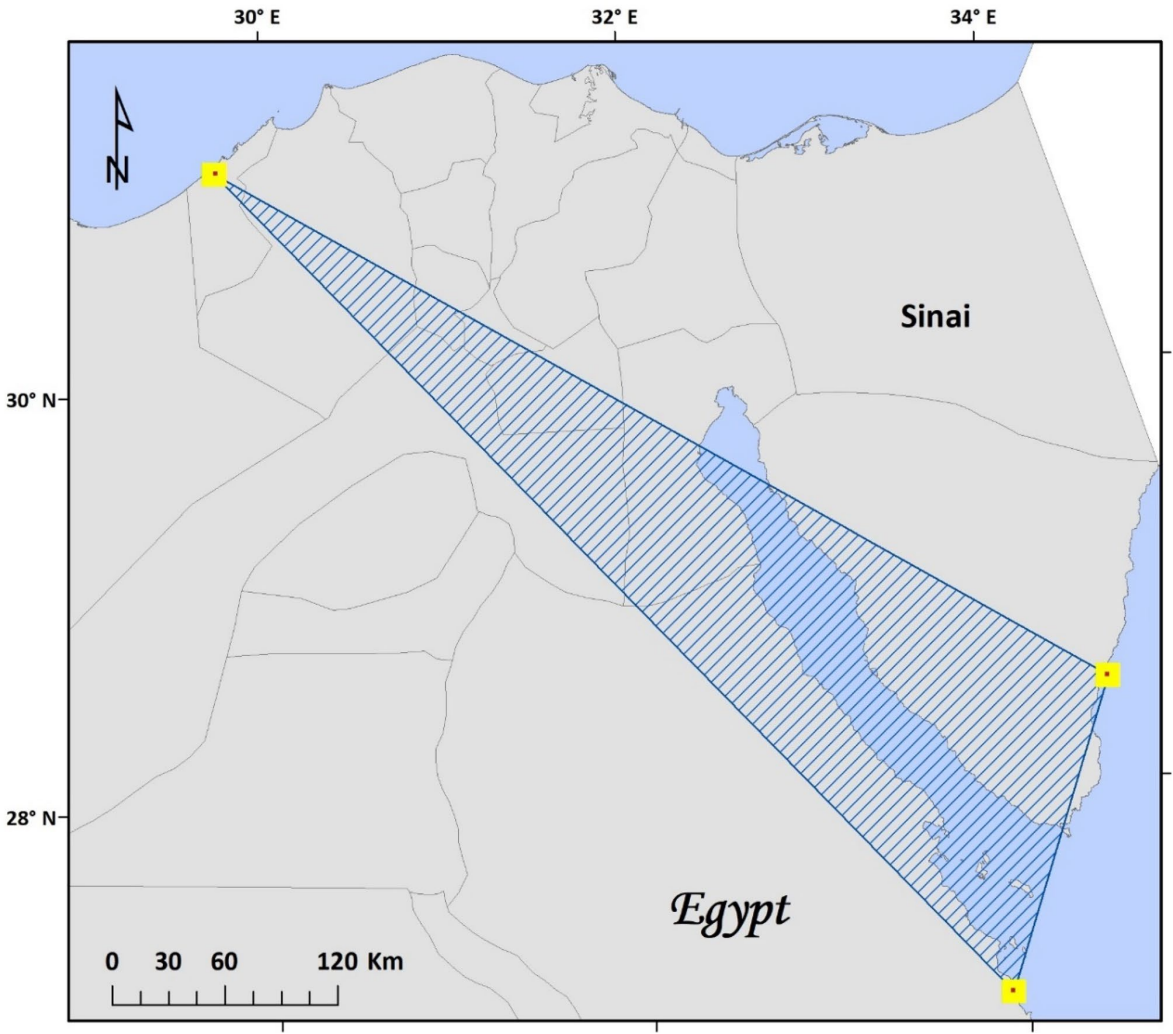
Distribution maps of *S. portulacastrum* in Egypt; yellow square are areas of observations, hatched polygon represents the known EOO as of 2022. The map was produced within the framework of ArcMap 10.1 GIS software package^74^.

The species increased its the area of occupancy (AOO) by 55% and extent of occurrence (EOO) by 17.5% from 2019 to 2020 at the study area (Table 5). At the national scale *S. portulacastrum* increased its AOO by 200% and EOO by more than 800% from 2019 to 2022 (Table 6; Fig. 4). The assessment of the expansion speed of the species at study area was estimated at 0.16 ha/year between 2018 and 2022, while it was revealed that it is expanding at faster rate at the national scale estimated at 600.9 ha/year.

**Table 5:** Estimates of expansion parameters for *S. portulacastrum* in the study area.

Year	EOO Convex hull area (ha)	AOO (ha)	Expansion speed (ha/year)
2018	1.89	0.51	0.16
2019	1.97	0.74	
2020	2.22	0.79	

The area of occupancy (AOO), extent of occurrence (EOO); and expansion speed.

**Table 6:** Estimates of expansion parameters for *S. portulacastrum* in Egypt.

Region of observation	Year	AOO (ha)	EOO convex hull area (ha)	Expansion speed (ha/year)
Northwest coast	2018	400	1.89	600.90
Northwest coast	2019	400	1.97	
Northwest coast	2020	400	2.22	
Northwest coast, South Sinai	2021	800	5303.91	
Northwest coast, Red Sea, South Sinai	2022	1200	4,673,290.0	

The area of occupancy (AOO), extent of occurrence (EOO); and expansion speed.

### Anatomy of the species

**Figure 5:**
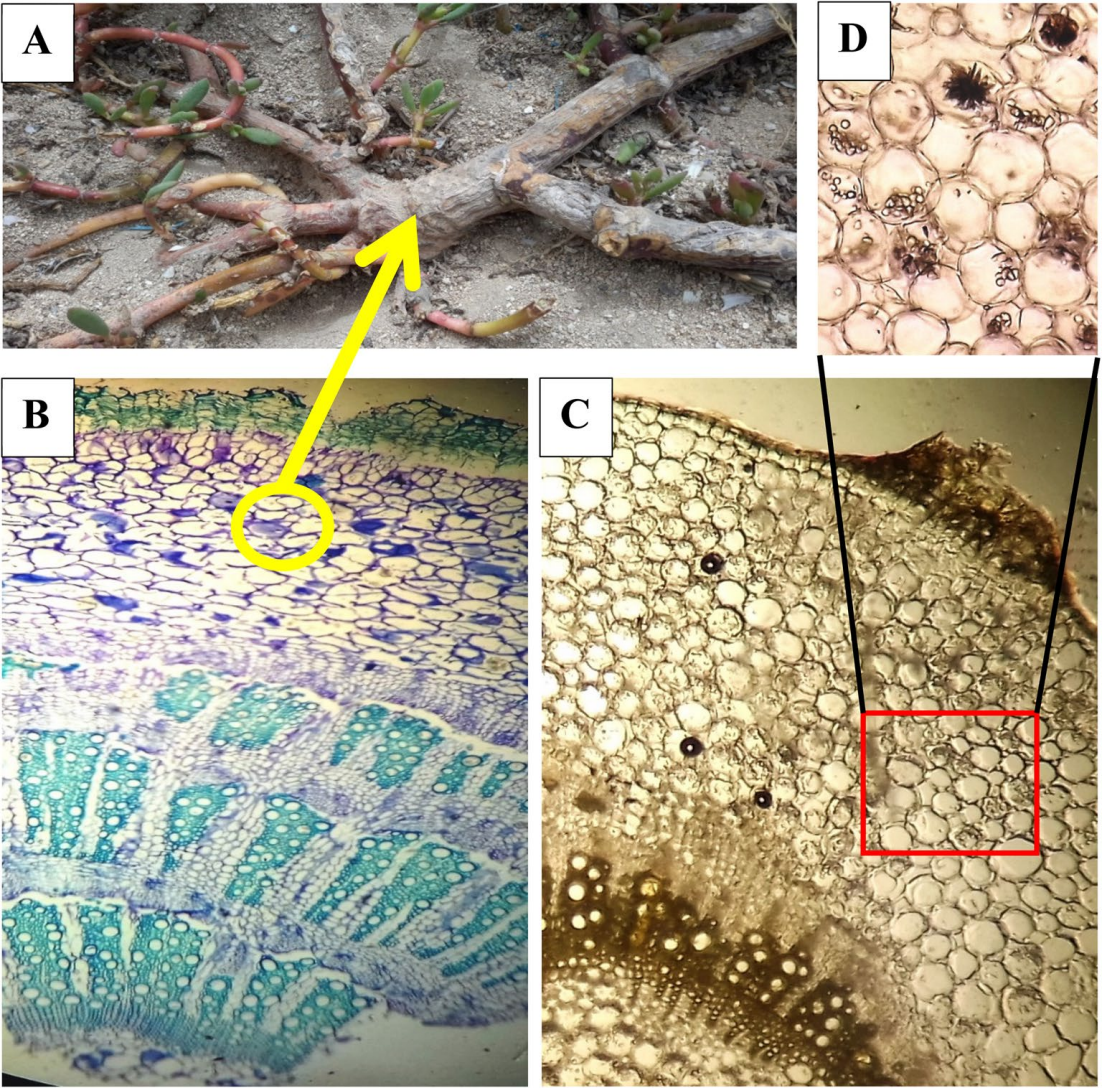
(**A**) The main stem and lateral branches of *S. portulacastrum* the yellow circle indicates a lateral branch. (**B**) Transverse section of lateral stem showing successive rings of vascular tissue. (**C**) Transverse section showing the periderm, lenticel and calcium oxalate crystals. (**D**) Enlarged part of the cortex showing calcium oxalate crystals (Druses).

The examined mature stems of *S. portulacastrum* were composed of successive rings of xylem in alternation with phloem (Fig. 5), and three to six successive rings of cambia. It is notable that the formation of vessels and phloem remains restricted to certain segments of the cambium. In the successive rings of the lateral meristem, small segments were differentiated into the vascular cambium that are giving rise to axial parenchyma, fibres and vessels to the inside, and secondary phloem to the outside. In certain parts, the cambial segments entirely were differentiated into derivatives leading to the disappearance of the cambial cells between the xylem and phloem. The epidermis is single layered made up of isodiametric cells and covered with thin cuticle that enclosed multi-layered parenchymatous cortex. Druses were present in the cortical region. Periderm and lenticels were also noticed in mature stems of *S. portulacastrum*.

### Germination and propagation

The plant exhibited a seed germination of 15% in petri-dishes and 13.3% in soil. The seeds were not subjected to any kind of treatment priorly. The germination of the seeds started after 10 days. *Sesuvium portulacastrum* can be propagated by seed and by rooted stem cuttings. The growth rate of the planted rooted stem cuttings was assessed, and it was observed that the planted segments did not take very long time to establish in the new substrate. It was also observed that the flowering buds in the planted segments reached maturity successfully to flowers shortly after it was planted. In February 2019, the cultivated mat reached about 6 m^2^ in ground cover with dimensions of 172 × 238 cm, and mean branch length of 170 ± 22.86 cm.

## Discussion

### Ecology of *S. portulacastrum* in the new habitat

*S. portulacastrum* was introduced from France to Egypt 10 years ago to private garden as an ornamental species (personal communication). A population of the species was recorded in Maruit region growing on saline sandy soils on the banks of an irrigation canal, which has been drilled about ten years ago, near Maruit Lake in the north-western coastal desert of Egypt. It is not clear whether the species was brought to the investigated region intentionally or accidently. Based on the field observation, the species is proliferating in the investigated site forming large mats of mean size up to nine square meters. These measures are higher than those recorded in its native habitats, based on the records in its native region it grows forming mats or patches up to two square meters or more in diameter^17^. The plant was found associated with common halophytic perennial species that inhabit sandy saline habitats in Egypt.

It appears that *S. portulacastrum* has the ecological tolerance that allows it to survive under various stress conditions. *S. portulacastrum* has not been recorded as part of the floral composition of the north-western coastal region at any previous study including all the study project that have conducted long term monitoring of the flora of the region (e.g.,^12–14,77^). The pattern of distribution of the species as it was recorded and illustrated in the produced maps indicates a random distribution, not a uniform regular distribution of the species in the region. Based on these observations and the monitoring conducted for the investigated site, it appears that the species is in the process of naturalization in the region. Naturalized species are those that have become established as a part of the plant life of a site that does not occur in their native range^78^. The plant must meet two conditions to be qualified as naturalized. First, the species must be alien (i.e., ‘exotic’, ‘introduced’, and ‘non-native’). Second, the plant must be capable of growing and producing a new generation without human interference (e.g., without irrigation, fertilizers, or pest control). That is, the plant turns into a wild species in its new region. Not all introduced plants species have the ecological tolerance to naturalize. Naturalized plant can either be beneficial or harmful in its new region that usually depends on the ecological settings of the area.

The species has the potential to proliferate in the investigated site, because it has been reported that it has the capability to grow and flourish in harsh conditions (salinity and drought). It grows in full sun and can tolerate both acidic and alkaline soils, in addition to its high drought tolerance^23^. Also, it is frequently reported as a pioneer species that has the capacity to establish itself in newly formed habitats and tolerate harsh condition^17^.

It has been reported that *S. portulacastrum* plays a significant role in coastal geomorphology as a dune initiator^79^. The plant can reproduce by sexual and asexual means^17^. Moreover, monitoring the species in the investigated site has revealed that the plant is flowering all year round, and can inhabit ruderal habitats. It was also, observed that the seeds may fall out of the remaining part of the fruit or remain on the plant after it dies.

### Characteristic of *S. portulacastrum* in the new habitat

The occurrence of secondary protective tissues (i.e., periderm and lenticels) noticed in the mature stems of *S. portulacastrum* indicated that this plant has been established in the investigated area long enough to produce secondary growth. The examination of the mature stems of *S. portulacastrum* indicated that the species has been introduced to the investigated area nearly six years ago, as was revealed by three successive rings of xylem alternating with phloem, composed of three to six successive rings of cambia.

The mature stem of *S. portulacastrum* lacked ray parenchyma; however, the occurrence of rays was reported by other studies^80,81^. The results revealed occurrence of initial stage of development of xylem rays. Rajput and Patil^82^ reported that in the case of *S. portulacastrum* secondary xylem remains rayless in the initial stage of secondary growth, but rays develop in the later stage of the secondary growth when the stem exceeds 20 mm in diameter.

The existence of druses in the cortex and pith is characteristic for *S. portulacastrum*^83^, which is attributed to many functions of which maintaining the ionic balance and preventing the accumulation of the toxic oxalates, storage of calcium in cells, and enhancing the mechanical support of the plant^83,84^.

The studied species produces seeds throughout the year and that shows a continuous germination pattern. However, it attains a low germination percentage in both soil and petri-dishes. Other studies have also reported that the species have a low germination percentage (e.g.,^85^). It seems that this might be attributed to the fact that seeds of *S. portulacastrum* require fluctuations in temperature and the exposure to light^45^. It was reported that the species germinates only in moist habitats^28^. The seeds that germinate near parent plants or under taller plants may not receive sufficient light or nutrients to survive^17^. Sauer^30^ inferred that the small seeds may be carried externally by shore birds. Also, seedling emergence could be inhibited when seeds are buried, this varies depending on the depth and the seed size. Germination can occur under a variety of salinity levels^45^.

*S. portulacastrum* can be propagated vegetatively using rooted stem cuttings. The plant roots easily from shoot fragments and stem cuttings. The species succeeded to establish itself in the new habitat with notably high growth rate. It is a low-maintenance plant, needing no irrigation or fertilizer and serious diseases or pests are not noticed. No information is available on the viability of cuttings after long term exposure to seawater. It is recommended that further experimental work be done on germination and seedling establishment under field conditions, to understand the way in which some factors regulate and limit species distribution along the different dune microhabitats. Further studies will also be needed to understand the factors determining the variability in the species germination response.

### Current distribution and rate of expansion

The revealed trend towards expansion in the species distribution in areas outside its native range as detected from the occurrence records of the species between 2018, and 2022 reflects the activities of introducing the species to new areas. The species is known to occur in the coastal areas of tropical Africa especially in areas characterized by hot and humid climate^86^, however the records recently detected out of the species native range, particularly in north Africa and west Asia. The role of climate change in the success of the establishment of the species in these new habitats need to be thoroughly investigated.

The local influence of an alien introduced species is a function of the extent it inhabits;^69,87^. The expansion speed based on the increase in the spatial presence, revealed the success of the introduced *S. portulacastrum* species in establishing itself in the new habitat. The rapid expansion speed by which the species increased its the area of occupancy (AOO) and extent of occurrence (EOO) at both local and the national scale indicated that the species has successfully established in the investigated regions and can be considered as naturalized species in the Egyptian flora. However, more investigations are needed on the dispersal mechanisms at the inhabited sites and how it can help in facilitating the spread and expansion of the species range. Due to the distinct physiological and molecular elasticity of *S. portulacastrum* that enables it to adapt and survive under various stress conditions^20–23^, the species is expected to spread in the future. Also, more explorations, surveys, and monitoring are needed to check for the occurrence of other populations of the species in the nation to contain its spread early if it was proven that it might have invasiveness potentiality.

Through obtaining the information describing the Extent of Occurrence (EOO) and Area of Occupancy (AOO) of the species over time, tracking further spread can easily be documented and mapped^71^. This information can help guide natural resources managers and decision-makers in taking appropriate actions. It should be noted that introduced species behave differently and there might be lag phase at the start of the expansion that vary depending on the species and the site-specific conditions^88^.

### Prospect of *S. portulacastrum* in Egypt

The development of mankind has reached the point at which a variety of new resources need to be taped in order to fill the basic need for food and feed, especially in the developing countries^89,90^. It is recommended to evaluate the economic viability and feasibility of utilizing *S. portulacastrum* for fodder and rangeland rehabilitation. Comparative analysis with conventional fodder highlights the plant’s elevated nutritional composition. This suggests a promising potential for utilizing the plant as a valuable fodder species. Some trials were conducted for cultivating the plant in the coastal salt marshes in North Sinai^91^. The feasibility of using the study species as fodder needs to be comprehensively assessed in Egypt. The restoration of desert habitats by planting indigenous or introduced species particularly ornamentals with high market value and wide range of ecological amplitude, could contribute to solving some environmental issues^37,44^.

The propagation of *S. portulacastrum* which tolerates salinity and drought in the salt marsh habitats in the desert could provide several ecological and economic benefits^33^. It could serve as an ornamental plant while providing ground cover to prevent erosion and enhance soil fixation^44^. As ornamental species it is characterized by its high growth rate, and it can easily spread to cover the ground with little water requirements^17^. Moreover, the plant has the potential as a source for extraction of pesticides, as it was reported that the insect moulting hormone 20-hydroxyecdysone has been isolated from *S. portulacastrum*^37^.

## Conclusions and recommendations

The study revealed that* S. portulacastrum* has successfully established in the northwestern coast of Egypt. Quantitative estimates of its spread rate and range of distribution have been estimated at the local and national scale. On the temporal and spatial scale, this assessment  will not only help in documenting the spread of *S. portulacastrum* but also in designing effective management strategy.

The species should be subjected to continuous monitoring to assess its behaviour in the wild habitats in Egypt. Further surveys should be pursued elsewhere in Egypt, to assess the species occurrence and rate of expansion. Detection of the spatial distribution and evaluation of the rate of expansion of *S. portulacastrum* based on the geospatial parameters, EOO and AOO can suitably be used as reliable estimates of the spread and naturalization potential of other invasive species. Furthermore, such information can be used as a baseline for comparing past and future changes in the distribution of alien and introduced species in any region. Systematic surveys conducted for floristic studies and collection of plant specimens for updating herbarium records can help in documenting the non-native flora. The chance of introductions of alien plants is high, due to the large proportion of agricultural materials being imported from other countries. It is recommended that concerned agencies initiate actions for systematically surveying any introduced species and pursuing measures that avoid alien species introduction. It is hoped that that the current study will highlight the importance of monitoring introduced species for early detection of invasion potential and minimizing risks to native plant diversity.

### Supplementary Information

Supplementary Figures.

## Data Availability

The datasets generated during and/or analysed during the current study are available from the corresponding author on reasonable request.
