# Supplementary material for: Naturalization and invasion potential of Sesuvium portulacastrum L. recorded as alien species in Egypt
Source: Sci Rep. 2024 Feb 7;14:3117. doi: 10.1038/s41598-024-53627-7 (PMC10850471; doi:10.1038/s41598-024-53627-7)
Supplement: Supplementary file 1 — Supplementary Figures. [file 41598_2024_53627_MOESM1_ESM.docx]

**Supplementary Material**

**
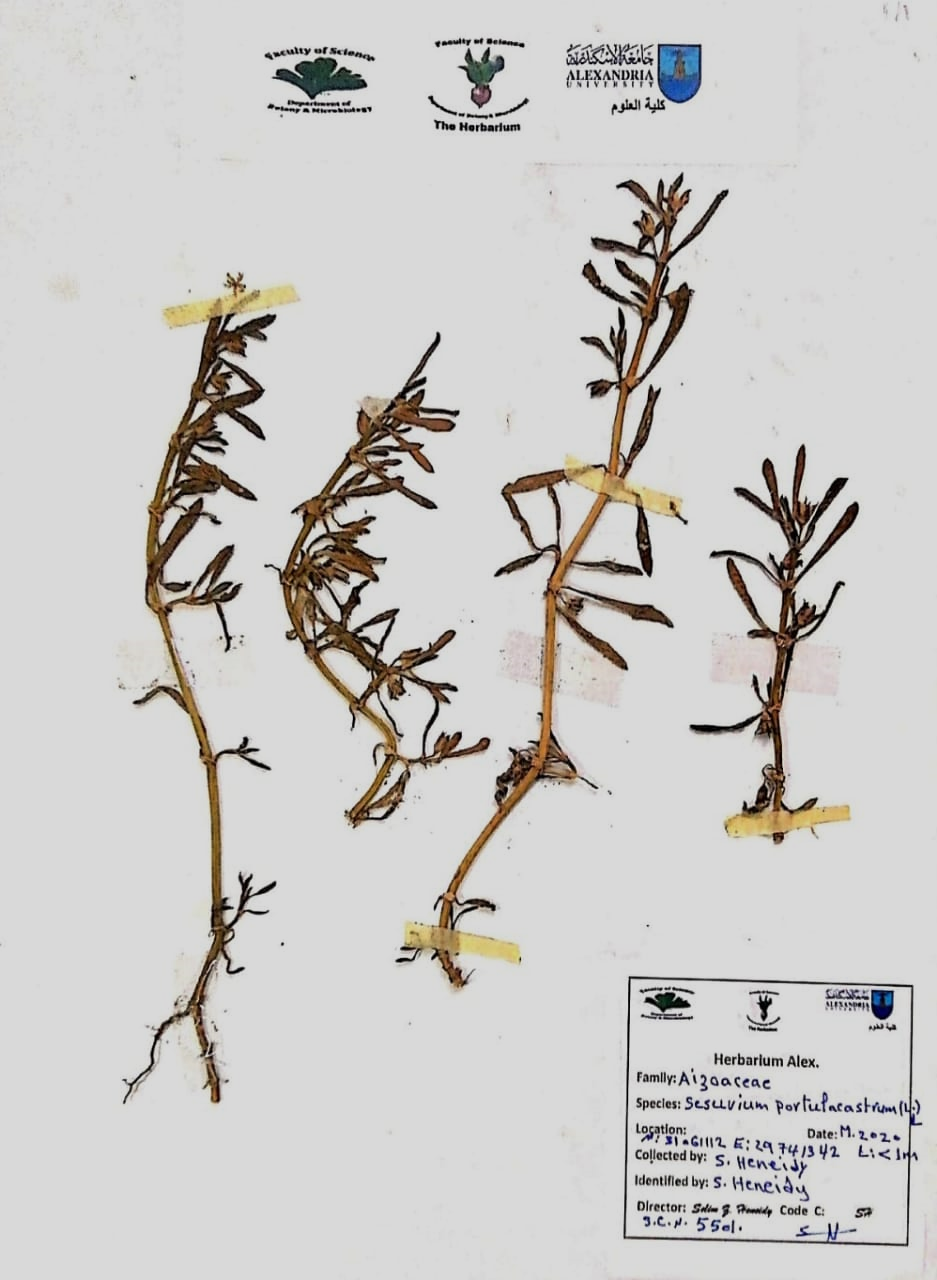
**

**Figure S1.** Herbarium sheet of *S. portulacastrum*, the voucher specimen is part of the Alexandria University Botanic Garden collection at the Herbarium of Alexandria University (ALEX). The specimens represent samples the population found near Maruit Lake at north-western coast of Egypt. Photo by S. Heneidy.


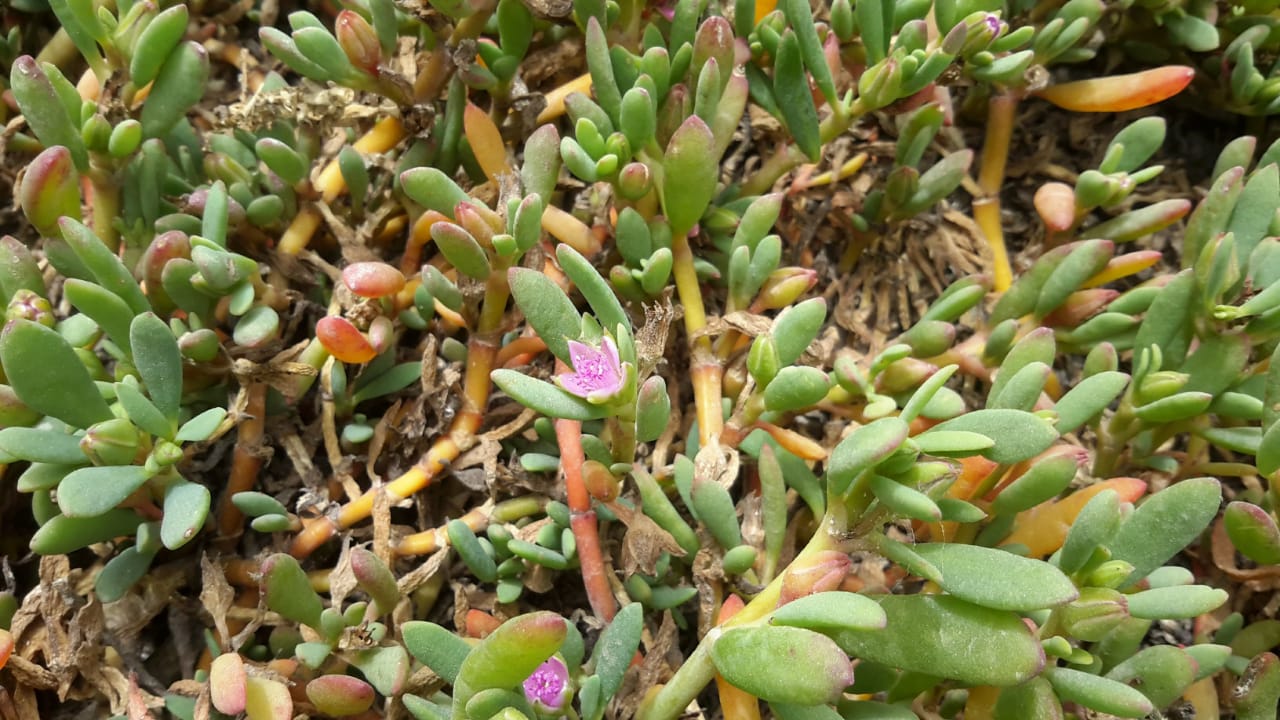


**Figure S2.** *Sesuvium portulacastrum* L. mat with purple flowers detected in the wild near Maruit Lake at northwestern coast of Egypt.


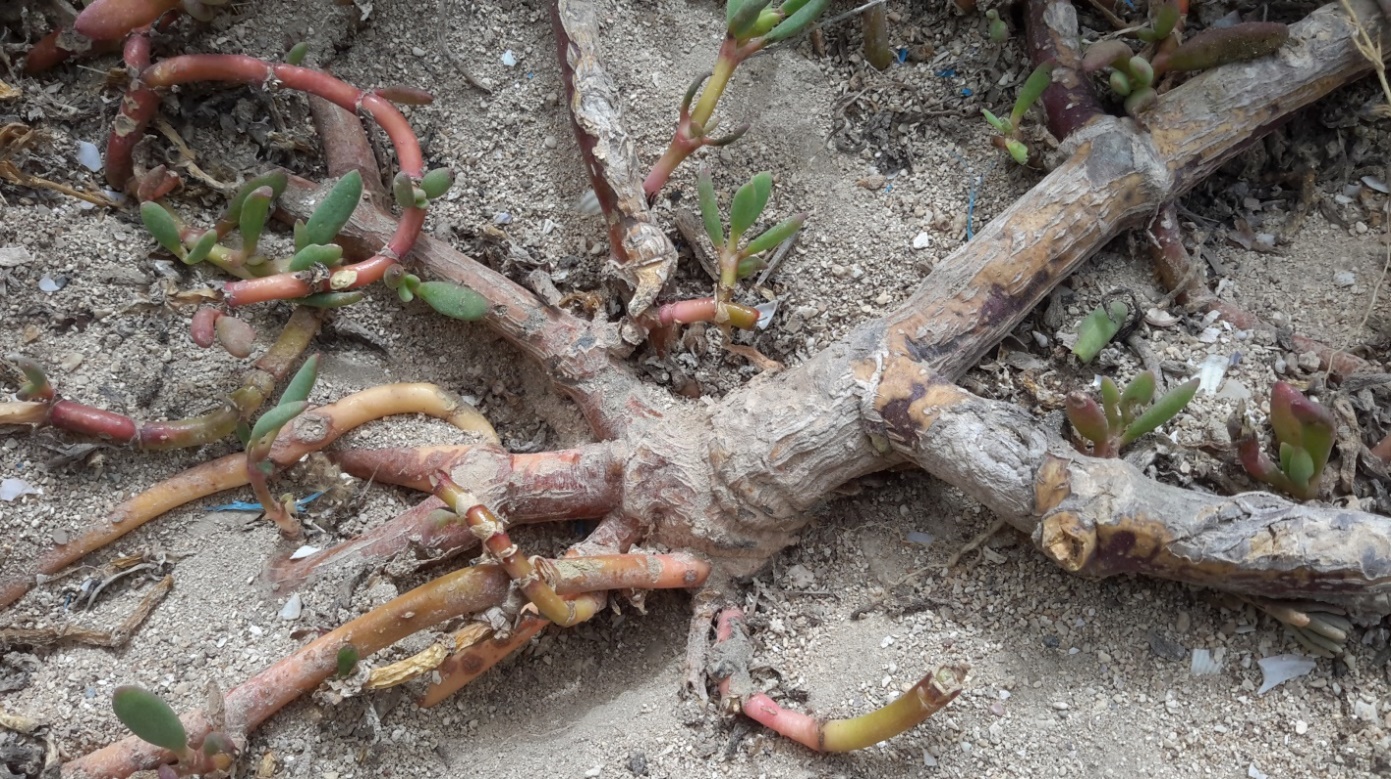


**Figure S3.** Mature stem of *S. portulacastrum* growing near Maruit Lake at northwestern coast of Egypt, photo by S. Heneidy.


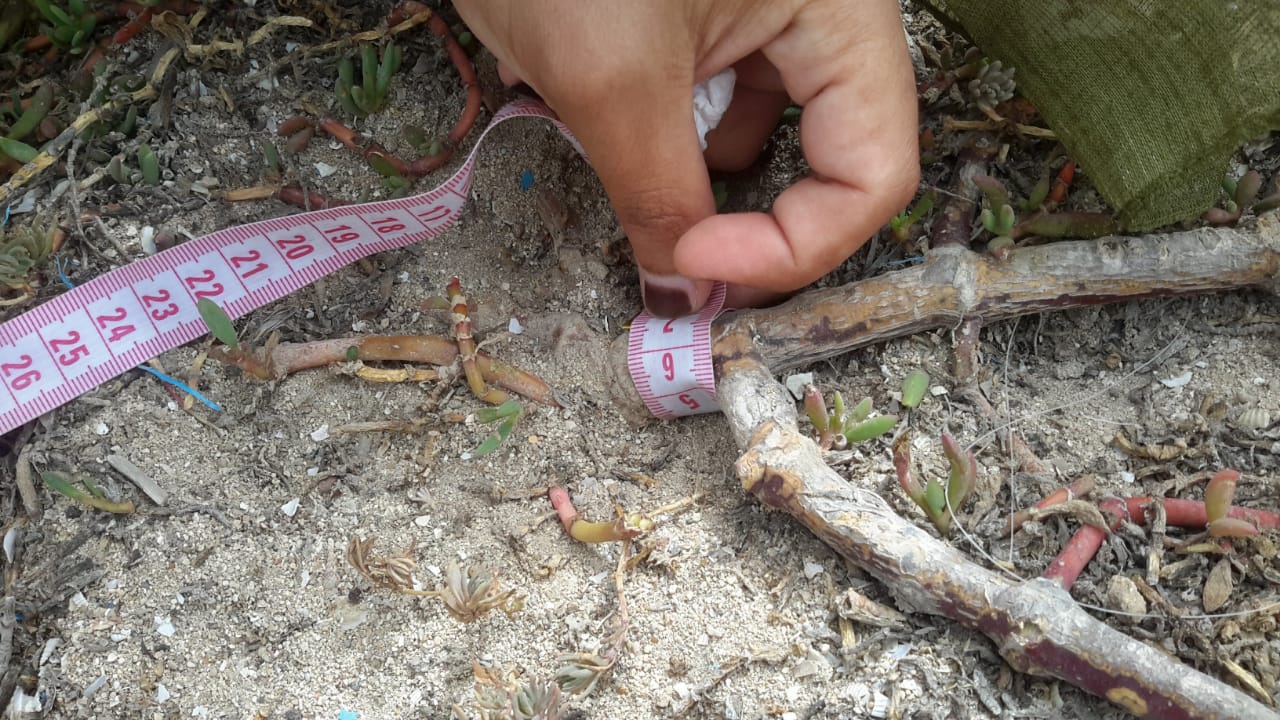


**Figure S4.** Measuring the diameter of a branch of *S. portulacastrum* growing near Maruit Lake at northwestern coast of Egypt, photo by S. Heneidy.


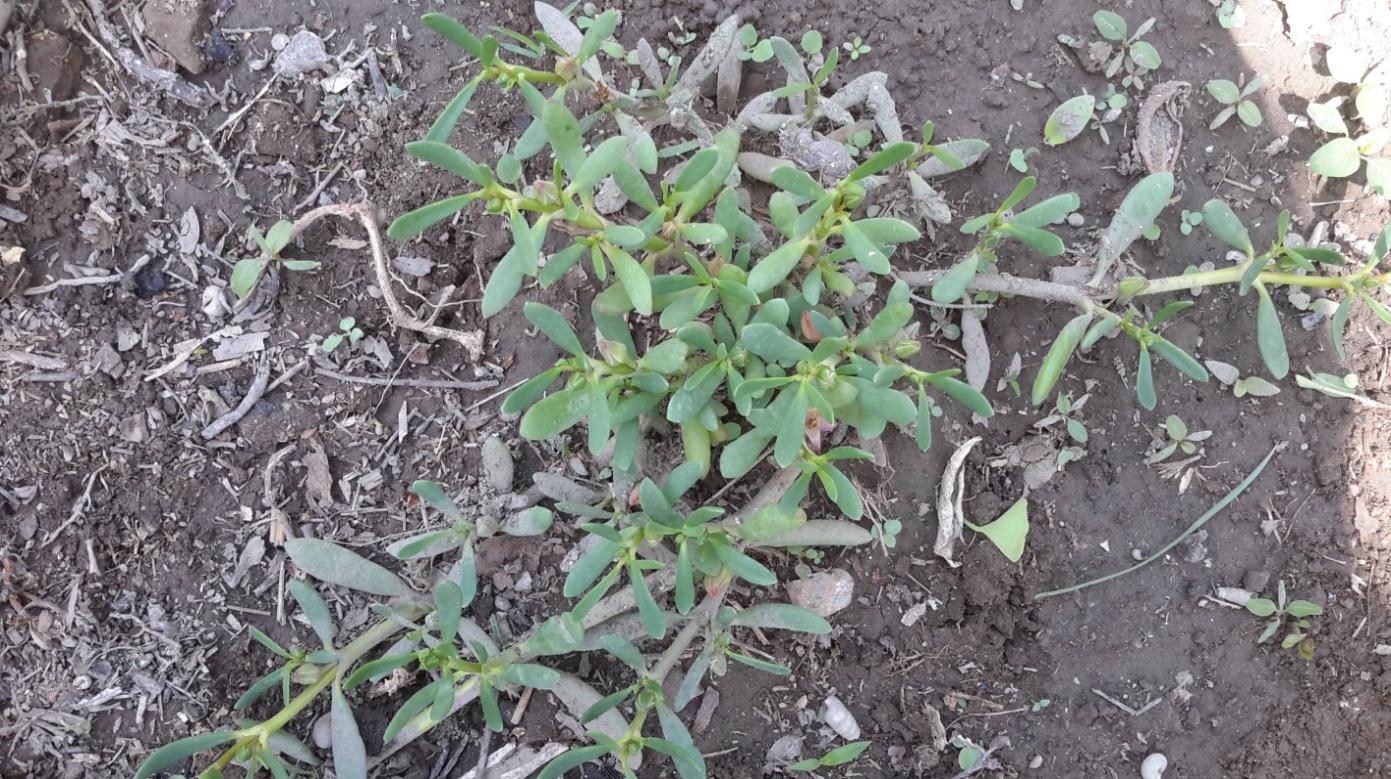


**Figure S5.** An individual of *S. portulacastrum* grown in the Alexandria University Botanic Garden. The Vegetative growth of segments brought from the population found near Maruit Lake at northwestern coast of Egypt were tested in the soil of the Alexandria University Botanic Garden, photo by S. Heneidy.
